# Supplementary material for: El Niño-Southern oscillation and under-5 diarrhea in Botswana
Source: Nat Commun. 2019 Dec 20;10:5798. doi: 10.1038/s41467-019-13584-6 (PMC6925142; doi:10.1038/s41467-019-13584-6)
Supplement: Supplementary file 1 — Supplementary Information [file 41467_2019_13584_MOESM1_ESM.pdf]

Supplementary Information

El Niño-Southern Oscillation and Under-5 Diarrhea in Botswana

Heaney et al.

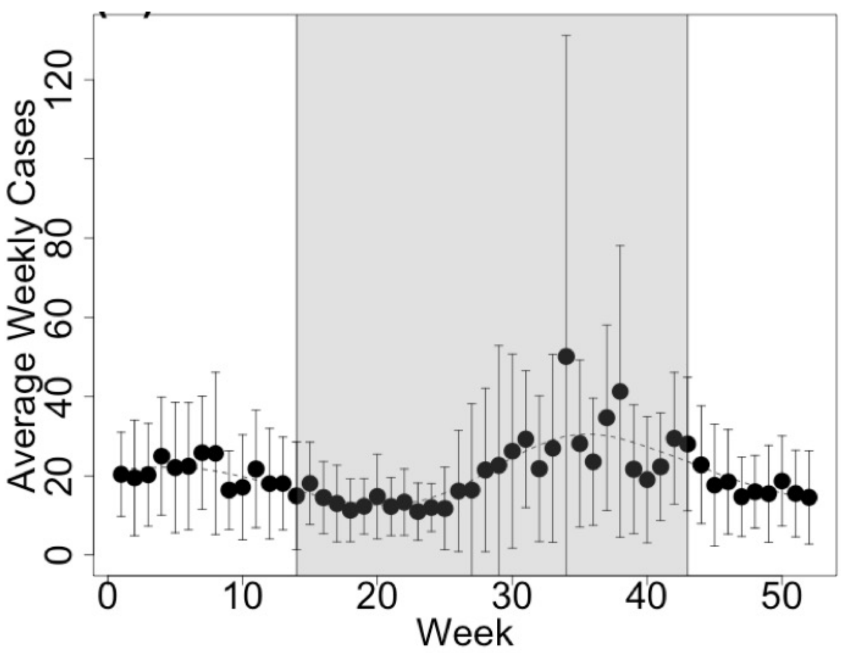

Supplementary Figure 1. Average weekly cases of under-5 diarrhea from January 2007 – July 2017 in Chobe District, Botswana. Dots represent the mean number of monthly cases and whiskers show 1 standard deviation in monthly cases across all years of data.

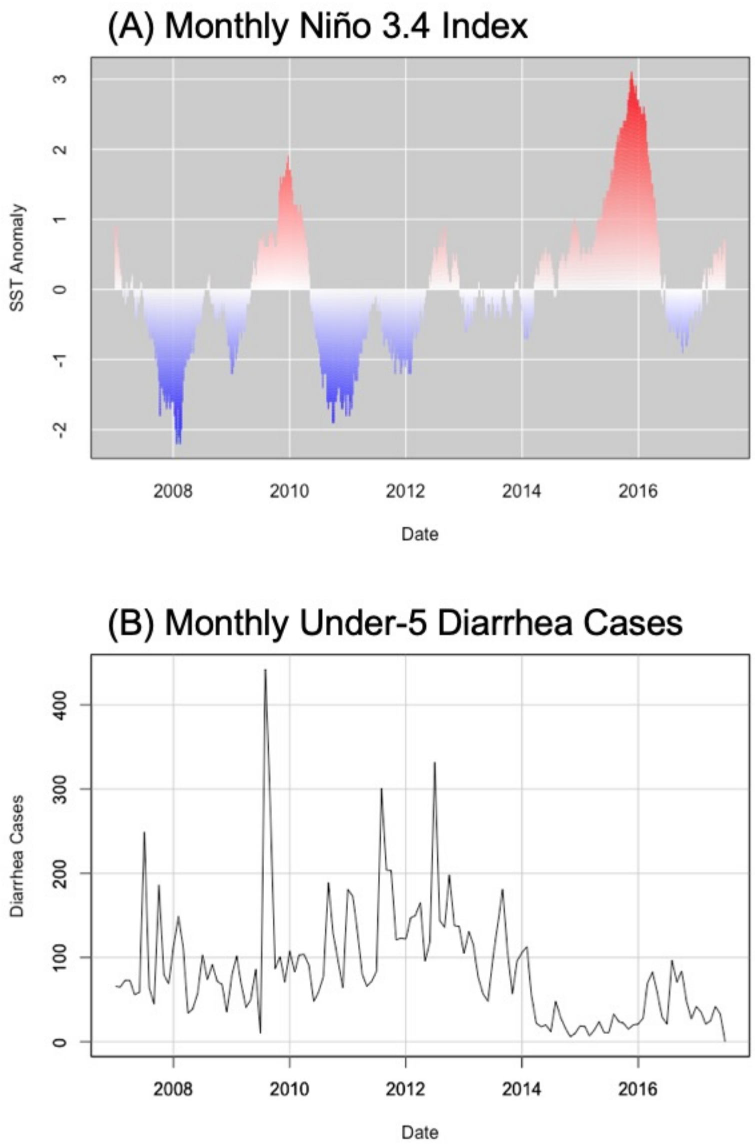

Supplementary Figure 2. Timeseries of monthly (A) Niño 3.4 SST anomalies in Kelvin and (B) under-5 diarrhea incidence in Chobe District, Botswana from January 2007 – July 2017. In panel (A) red shows warmer than average Niño 3.4 SST anomalies and blue shows cooler than average Niño 3.4 SST anomalies.

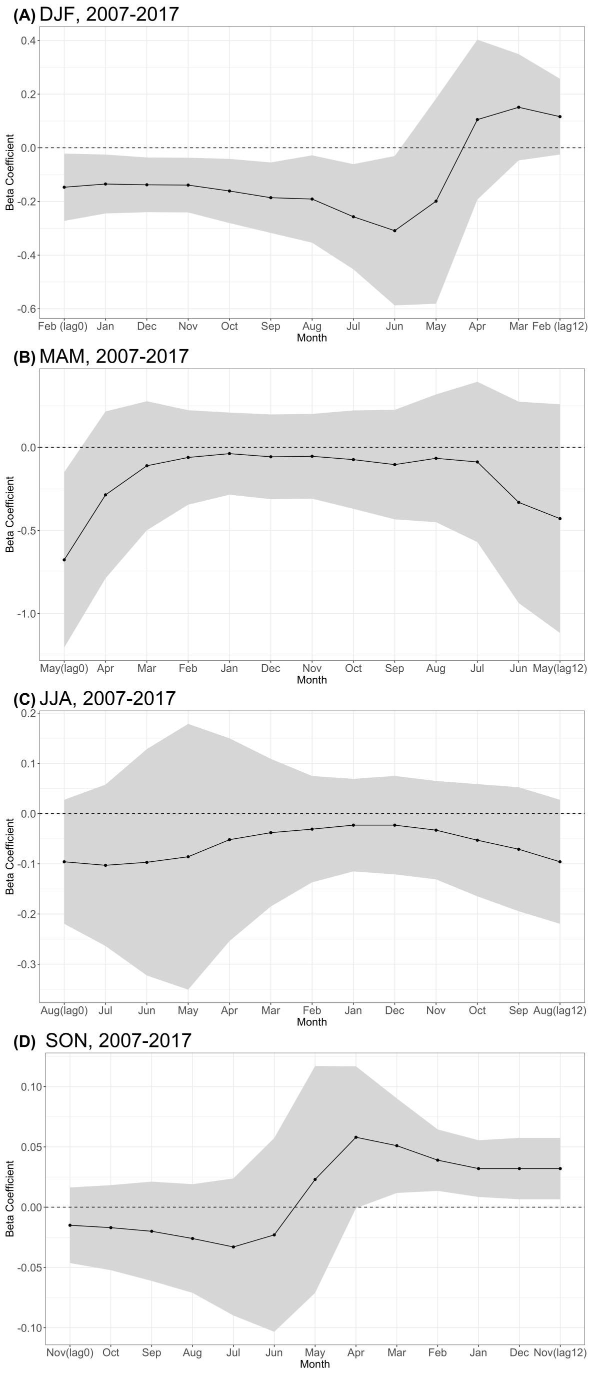

Supplementary Figure 3. Coefficient estimates and 95% confidence intervals for the association between Niño 3.4 and average Chobe River height in (A) DJF, (B) MAM, (C) JJA, and (D) SON during 2007-2017. The x-axis represents the monthly Niño 3.4 estimate during or before the corresponding season. The beta coefficient represents the change in seasonal Chobe River height in meters associated with a 1K increase in Niño 3.4.

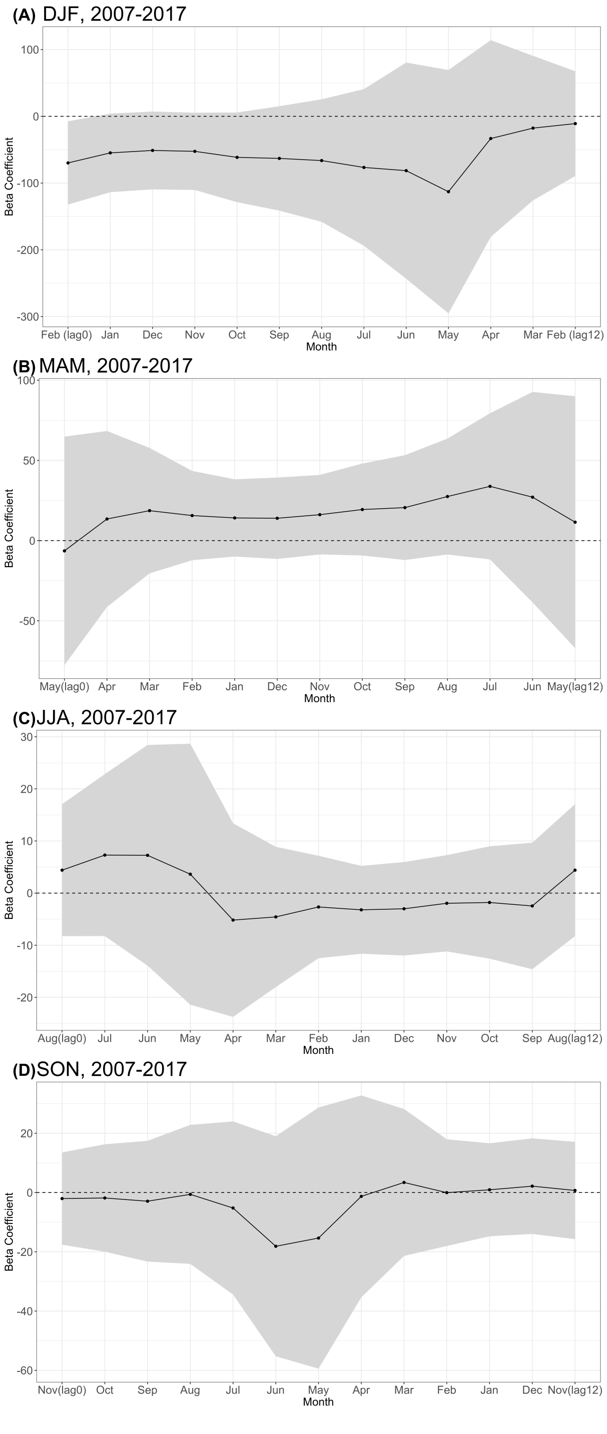

Supplementary Figure 4. Coefficient estimates and 95% confidence intervals for the association between Niño 3.4 and total Chobe District rainfall in (A) DJF, (B) MAM, (C) JJA, and (D) SON during 2007-2017. The x-axis represents the monthly Niño 3.4 estimate during or before the corresponding season. The beta coefficient represents the change in seasonal Chobe District rainfall in millimeters associated with a 1K increase in Niño 3.4.

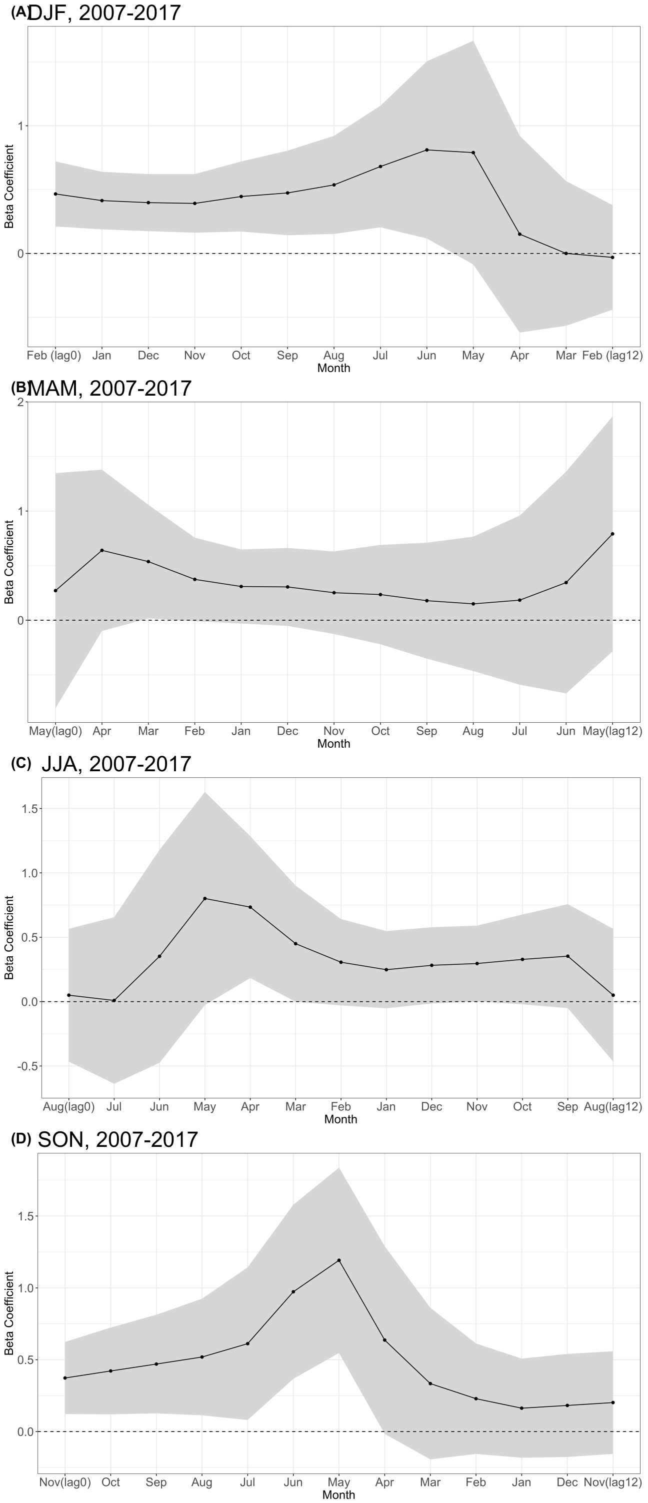

Supplementary Figure 5. Coefficient estimates and 95% confidence intervals for the association between Niño 3.4 and average Chobe District minimum temperature in (A) DJF, (B) MAM, (C) JJA, and (D) SON during 2007-2017. The x-axis represents the monthly Niño 3.4 estimate during or before the corresponding season. The beta coefficient represents the change in Chobe District minimum temperature in Celsius associated with a 1K increase in Niño 3.4.

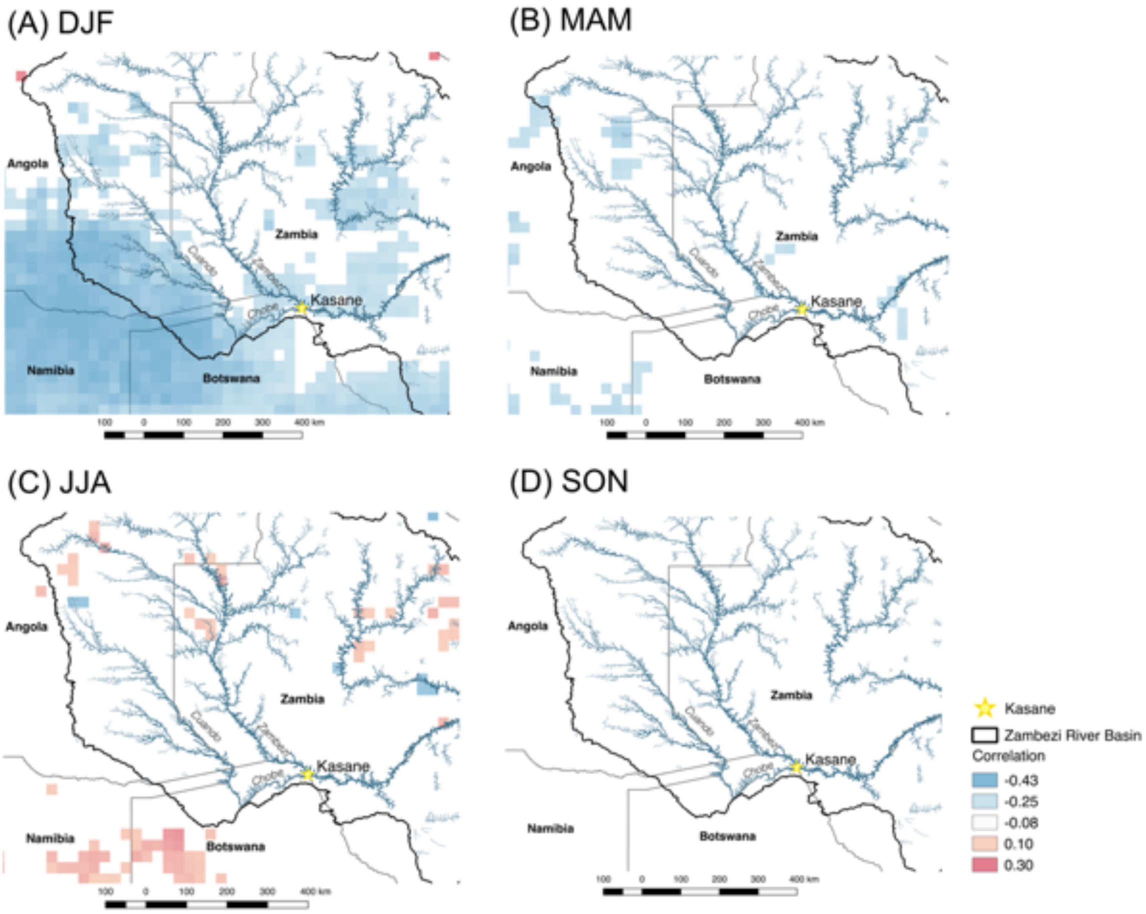

Supplementary Figure 6. Correlations between monthly Niño 3.4 and TRMM rainfall during 1998-2015 for (A) December-February, (B) March-May, (C) June-August, and (D) September-November. Blue represents negative correlations and red represents positive correlations. The gold star locates Kasane, which is the largest town in Chobe District, and the black line outlines the Zambezi River Basin. Only correlations statistically significant at $p<0.05$ are shown on the map.

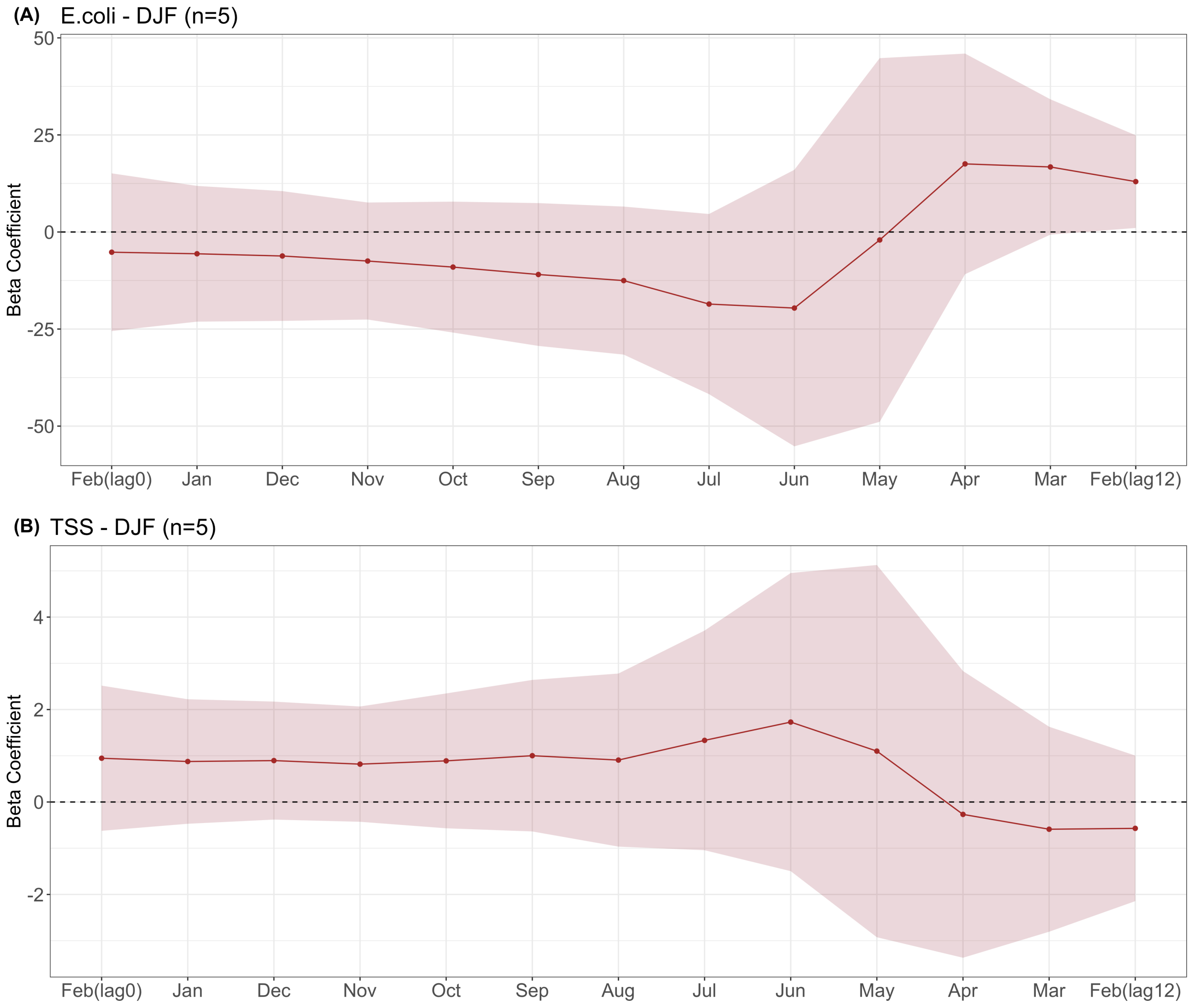

Supplementary Figure 7. Associations between Niño 3.4 and water quality. Coefficient estimates show the estimated change in (A) *E. coli* (count/milliliter) and (B) Total Suspended Solids (mg/L) associated with a 1K increase in Niño 3.4 during DJF. Water quality measures are mean measurements taken throughout DJF, and Niño 3.4 is lagged 0-12 months before the DJF season. The x-axis represents the monthly lagged Niño 3.4 predictor and the y-axis represents regression beta coefficients and 95% confidence intervals. The sample size for each beta coefficient estimation was 5 seasons (n=5).

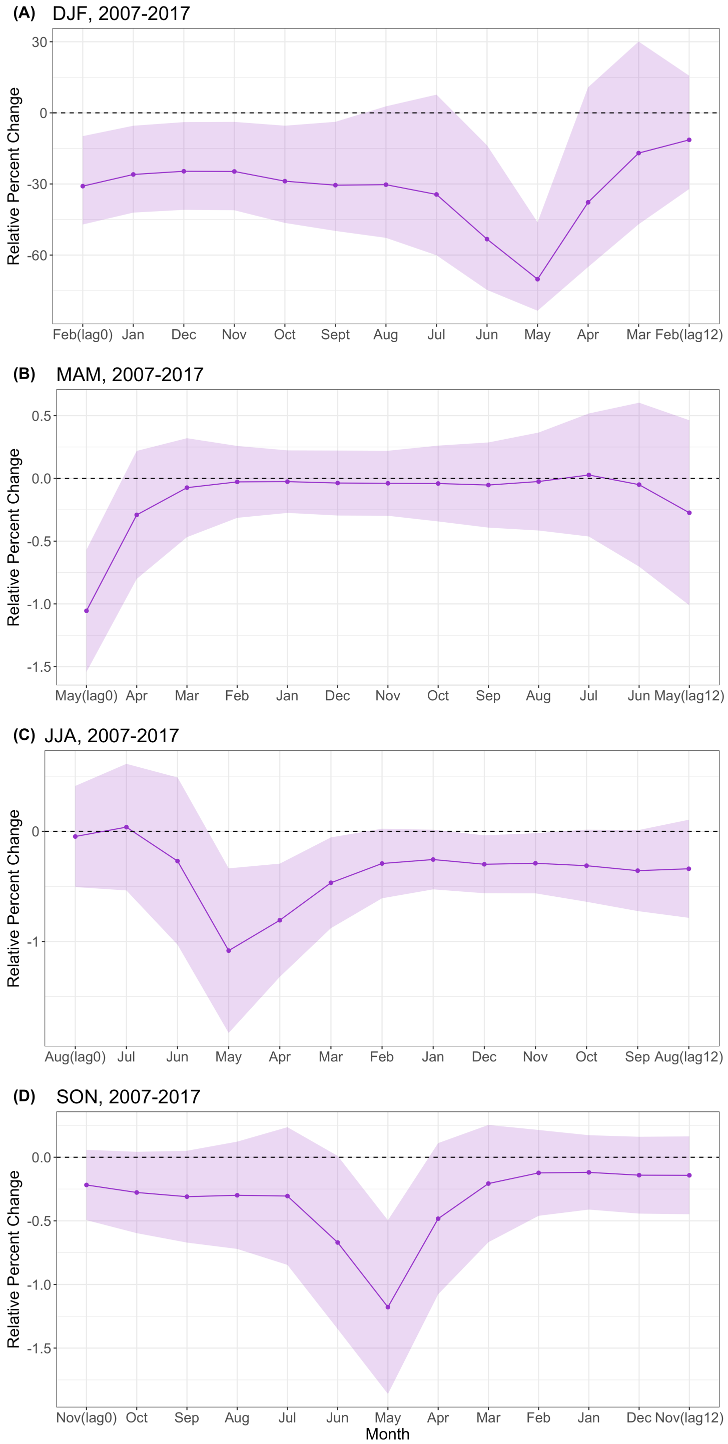

Supplementary Figure 8. Coefficient estimates and 95% confidence intervals for association between Niño 3.4 and total under-5 diarrhea cases in (A) DJF, (B) MAM, (C) JJA, and (D) SON. The x-axis represents the monthly Niño 3.4 estimate during or before the corresponding season. Estimates are shown using all years of data (2007-2017). The y-axis shows the relative percent change (and 95% confidence interval) in under-5 diarrhea incidence associated with 1K increase in Niño 3.4.

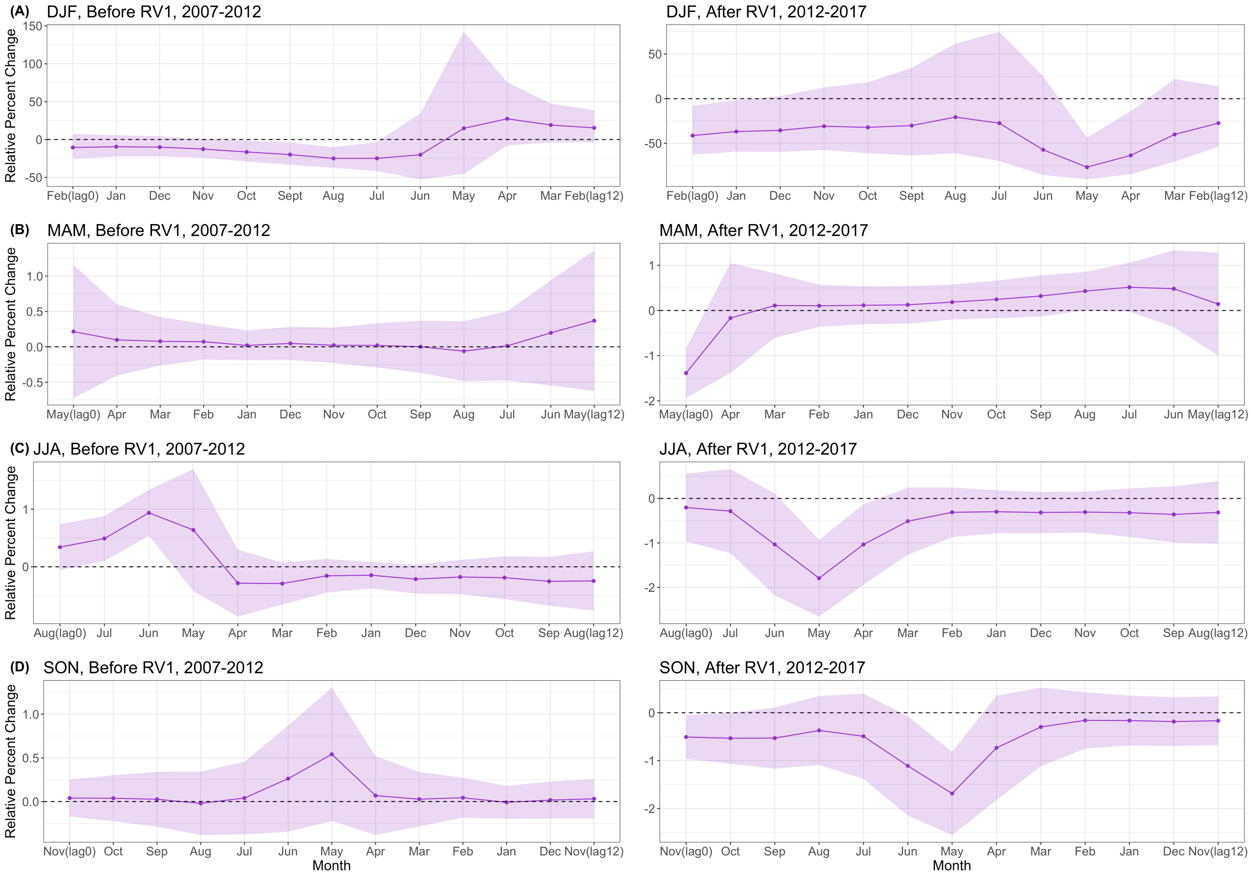

Supplementary Figure 9. Coefficient estimates and 95% confidence intervals for associations between Niño 3.4 and total under-5 diarrhea cases in (A) DJF, (B) MAM, (C) JJA, and (D) SON before and after the rotavirus vaccine (RV1) introduction. The x-axis represents the monthly Niño 3.4 estimate during or before the corresponding season. The y-axis shows the relative percent change (and 95% confidence interval) in under-5 diarrhea incidence associated with 1K increase in Niño 3.4. Estimates are shown using data collected before the RV1 rollout (2007-2012) and data collected after the RV1 rollout (2012-2017).

Analyses Using the Multivariate ENSO Index (MEI)

Supplementary Table 1. Correlations between MEI, environmental variables, and under-5 diarrhea.

	Diarrhea Anomalies	Rainfall Anomalies	Chobe River Height Anomalies	Minimum Temperature Anomalies
MEI lag 0	-0.354 (p<0.001)	-0.067 (p=0.454)	-0.318 (p<0.001)	0.397 (p<0.001)
MEI lag 1	-0.354 (p<0.001)	-0.059 (p=0.511)	-0.254 (p=0.005)	0.426 (p<0.001)
MEI lag 2	-0.343 (p<0.001)	-0.082 (p=0.362)	-0.203 (p=0.026)	0.419 (p<0.001)
MEI lag 3	-0.361 (p<0.001)	-0.107 (p=0.236)	-0.173 (p=0.059)	0.402 (p<0.001)

Pearson's correlations between monthly MEI lagged 0-3 months, and monthly anomalies of under-5 diarrhea, and environmental variables. P-values were calculated using two-sided Pearson's correlation test with alpha of 0.05.

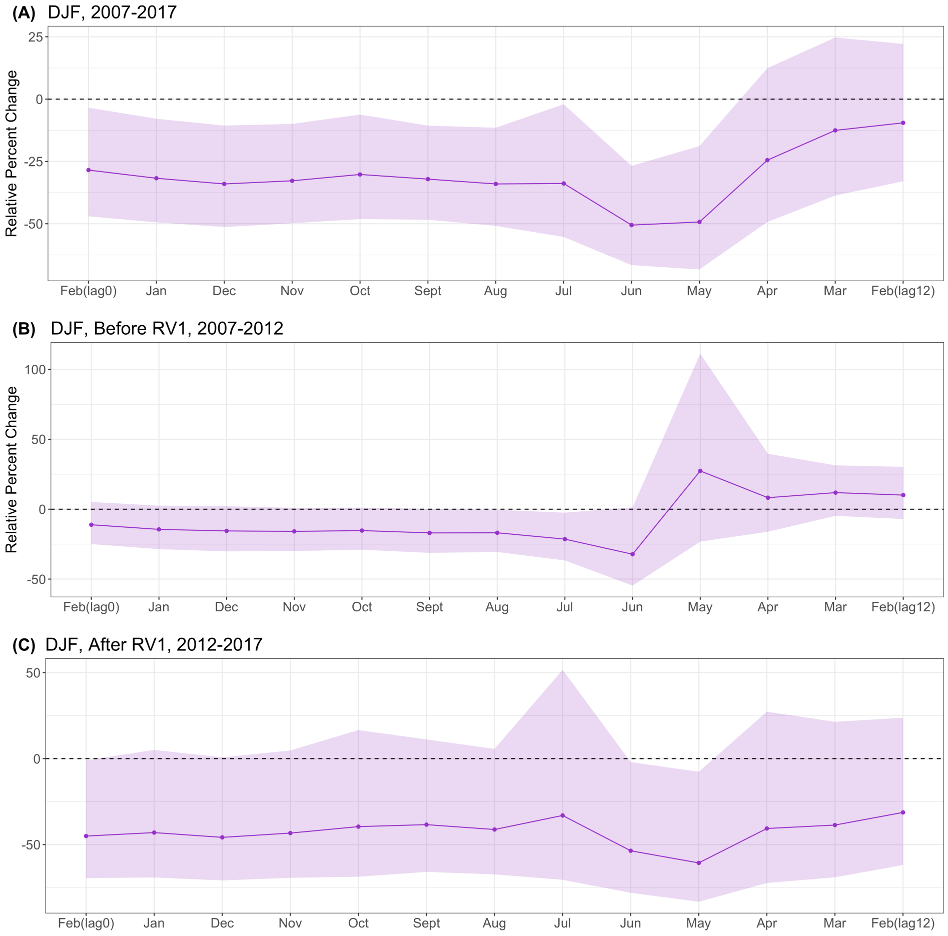

Supplementary Figure 10. December, January, February under-5 diarrhea associated with MEI. The y-axis shows the estimated percent change and 95% confidence intervals in DJF diarrhea incidence associated with 1K increase in MEI. The x-axis represents the month of MEI predictor, which is during or before DJF. Estimates are shown using (A) all of the data (2007-2017), (B) using only data collected before the rotavirus vaccine rollout (Dec. 2007- Feb. 2012), and (C) using only data collected after the rotavirus vaccine rollout (Dec. 2012- Feb. 2017).

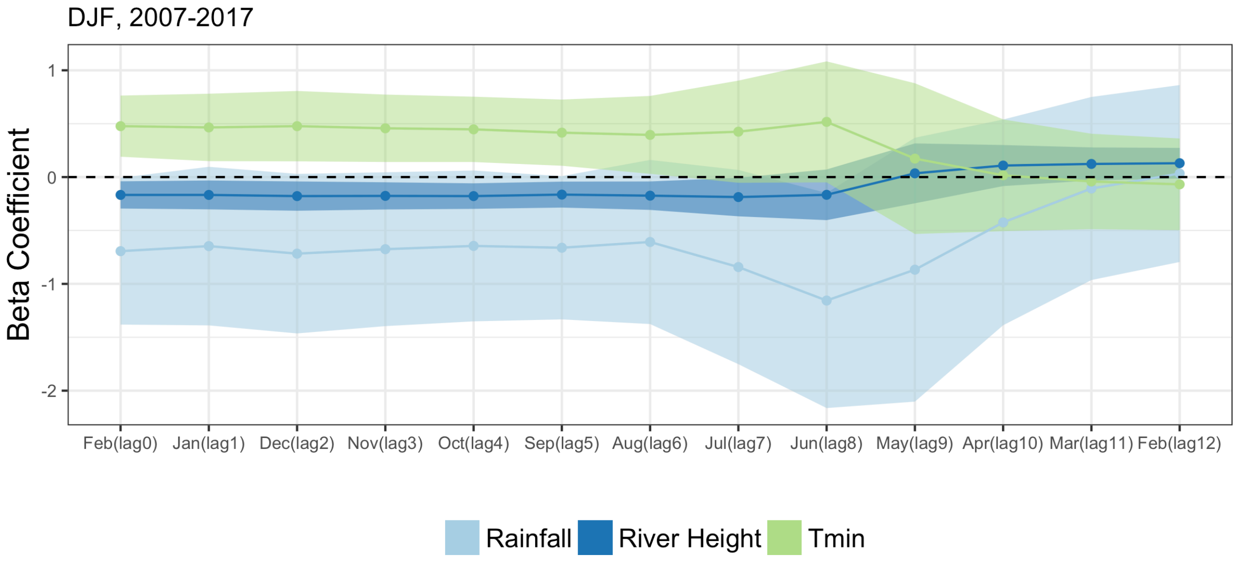

Supplementary Figure 11. December, January, February associations between MEI and environmental variables. Beta coefficients with 95% confidence intervals are shown from regressions predicting total rainfall (light blue, in 100s of millimeters/K), average Chobe River height (dark blue, in meters/K), and average minimum temperature (green, in degrees Celsius/K). Environmental outcomes in DJF were predicted using MEI lagged 0-12 months. The corresponding February from DJF season is lag 0, and the previous February is lag 12. Beta coefficients represent the change in the outcome (in 100s of millimeters for rainfall, meters for river height, and degrees Celsius for temperature) associated with a 1K increase in MEI. Regressions were run using data from 2007-2017.

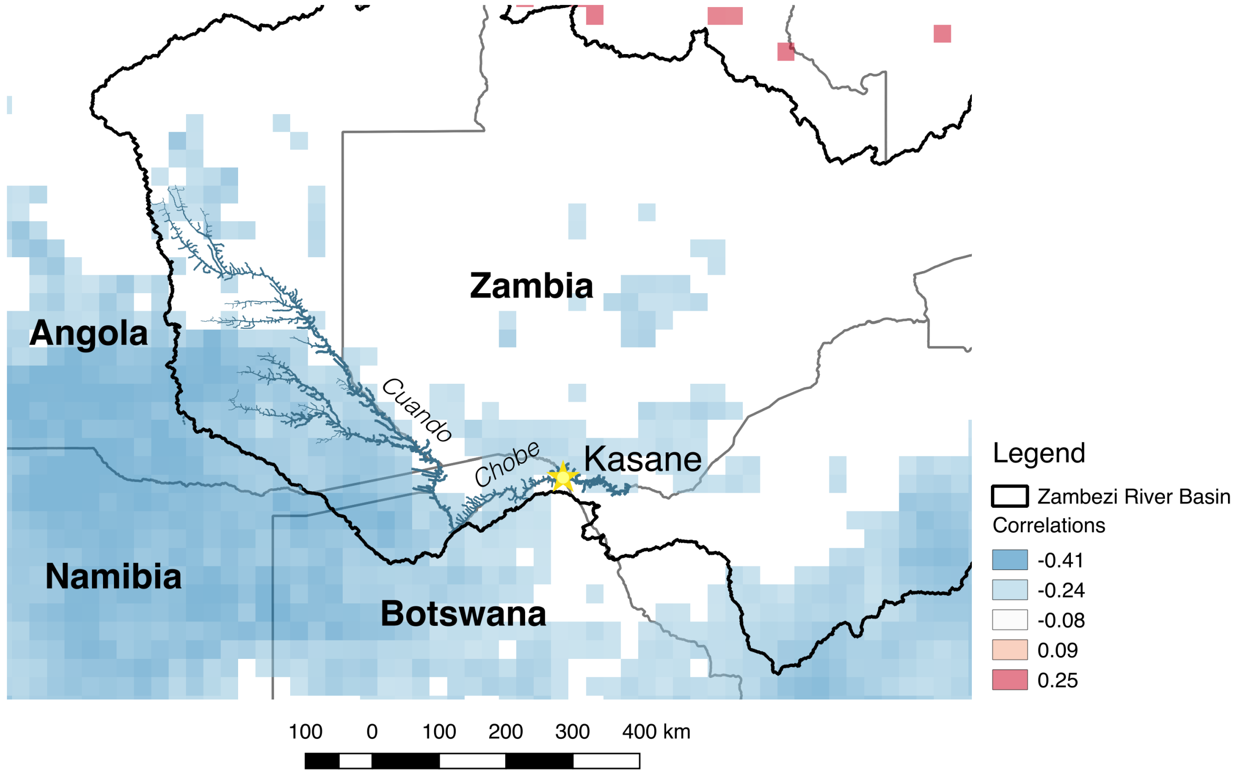

Supplementary Figure 12. Correlations between MEI and TRMM regional rainfall in December, January, February from 1998-2015. Only correlations statistically significant at $p<0.05$ are shown on the map. Blue represents negative correlations and red represents positive correlations. The gold star locates Kasane, which is the largest town in Chobe District, and the black line outlines the Zambezi River Basin.

Analyses Using the Southern Oscillation Index (SOI)

Supplementary Table 2. Correlations between SOI, environmental variables, and under-5 diarrhea.

	Diarrhea Anomalies	Rainfall Anomalies	Chobe River Height Anomalies	Minimum Temperature Anomalies
SOI lag 0	0.340 (p<0.001)	0.103 (p=0.248)	0.336 (p<0.001)	-0.279 (p=0.002)
SOI lag 1	0.340 (p<0.001)	0.098 (p=0.277)	0.290 (p=0.001)	-0.270 (p=0.002)
SOI lag 2	0.319 (p<0.001)	0.078 (p=0.388)	0.275 (p=0.002)	-0.237 (p=0.008)
SOI lag 3	0.258 (p<0.004)	0.126 (p=0.163)	0.301 (p<0.001)	-0.215 (p=0.017)

Pearson’s correlations between monthly SOI lagged 0-3 months, and monthly anomalies of under-5 diarrhea, and environmental variables. P-values were calculated using two-sided Pearson’s correlation test with alpha of 0.05.

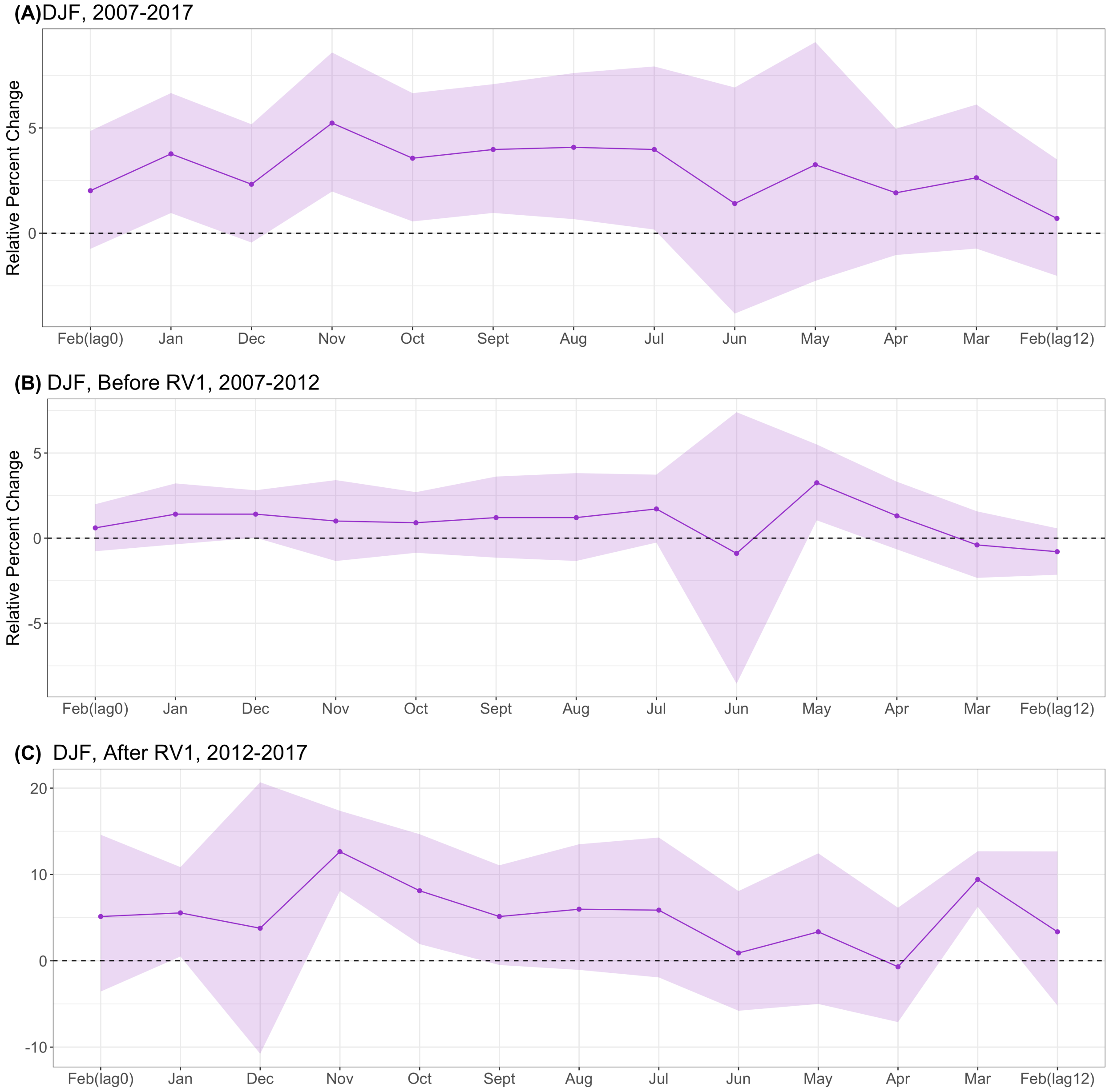

Supplementary Figure 13. December, January, February under-5 diarrhea associated with SOI. The y-axis shows the estimated percent change and 95% confidence intervals in DJF diarrhea incidence associated with 1K increase in SOI SST anomalies. The x-axis represents the month of SOI predictor, which is during or before DJF. Estimates are shown using (A) all of the data (2007-2017), (B) using only data collected before the rotavirus vaccine rollout (Dec. 2007- Feb. 2012), and (C) using only data collected after the rotavirus vaccine rollout (Dec. 2012- Feb. 2017).

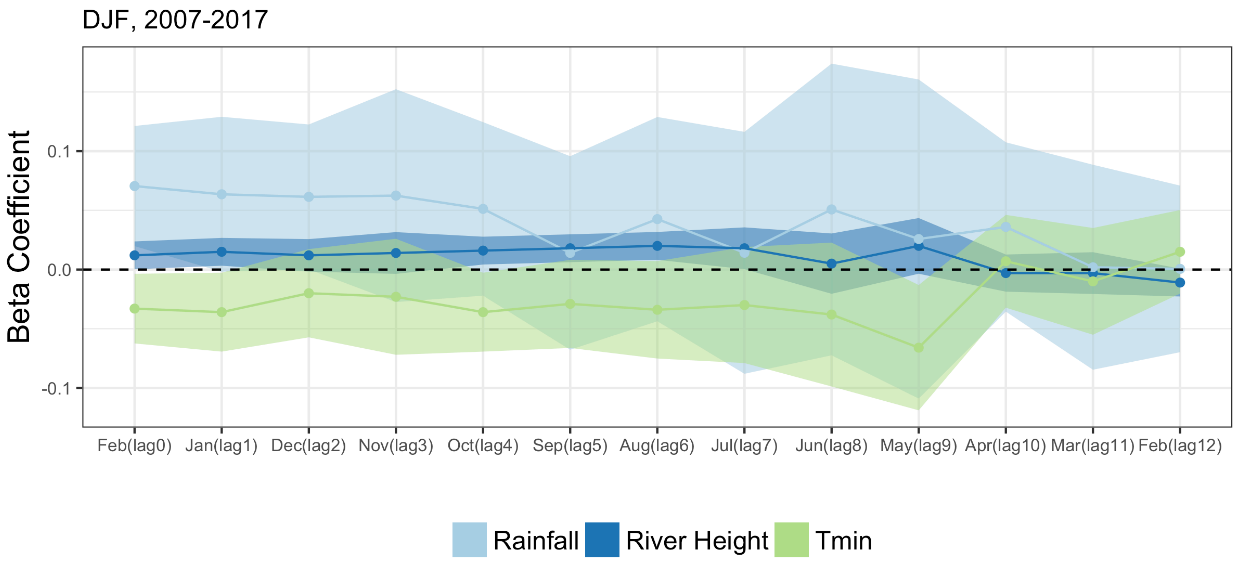

Supplementary Figure 14. December, January, February associations between SOI and environmental variables. Beta coefficients with 95% confidence intervals are shown from regressions predicting total rainfall (light blue, in 100s of millimeters/K), average Chobe River height (dark blue, in meters/K), and average minimum temperature (green, in degrees Celsius/K). Environmental outcomes in DJF were predicted using SOI lagged 0-12 months. The corresponding February from DJF season is lag 0, and the previous February is lag 12. Beta coefficients represent the change in the outcome (in 100s of millimeters for rainfall, meters for river height, and degrees Celsius for temperature) associated with a 1K increase in SOI. Regressions were run using data from 2007-2017.

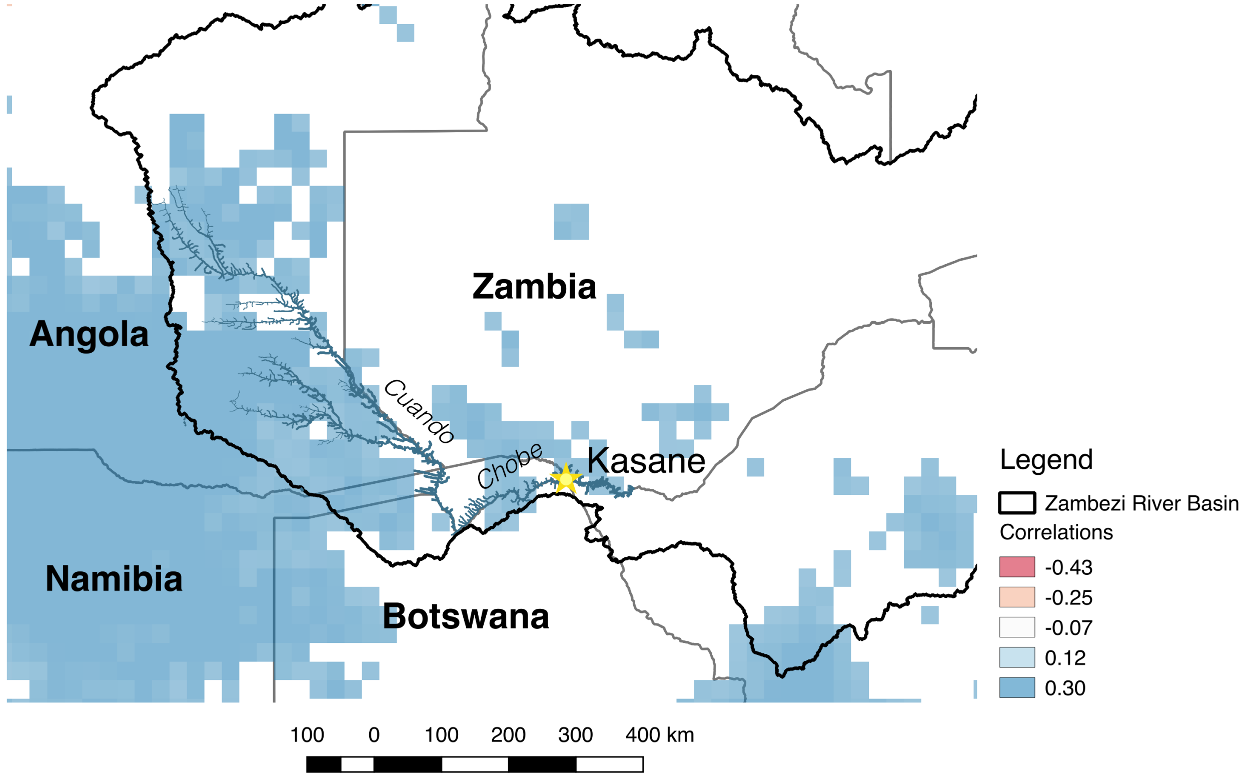

Supplementary Figure 15. Correlations between SOI and TRMM regional rainfall in December, January, February from 1998-2015. Only correlations statistically significant at $p<0.05$ are shown on the map. Red represents negative correlations and blue represents positive correlations. The gold star locates Kasane, which is the largest town in Chobe District, and the black line outlines the Zambezi River Basin.

Analyses Using the Niño 4 SST Anomalies

Supplementary Table 3. Correlations between Niño 4, environmental variables, and under-5 diarrhea.

	Diarrhea Anomalies	Rainfall Anomalies	Chobe River Height Anomalies	Minimum Temperature Anomalies
Niño 4 lag 0	-0.415 (p<0.001)	-0.103 (p=0.250)	-0.322 (p<0.001)	0.411 (p<0.001)
Niño 4 lag 1	-0.415 (p<0.001)	-0.123 (p=0.168)	-0.275 (p=0.002)	0.435 (p<0.001)
Niño 4 lag 2	-0.400 (p<0.001)	-0.118 (p=0.188)	-0.227 (p=0.012)	0.419 (p<0.001)
Niño 4 lag 3	-0.388 (p<0.001)	-0.097 (p=0.283)	-0.182 (p=0.046)	0.407 (p<0.001)

Pearson's correlations between monthly Niño 4 SST anomalies lagged 0-3 months, and monthly anomalies of under-5 diarrhea, and environmental variables. P-values were calculated using two-sided Pearson's correlation test with alpha of 0.05.

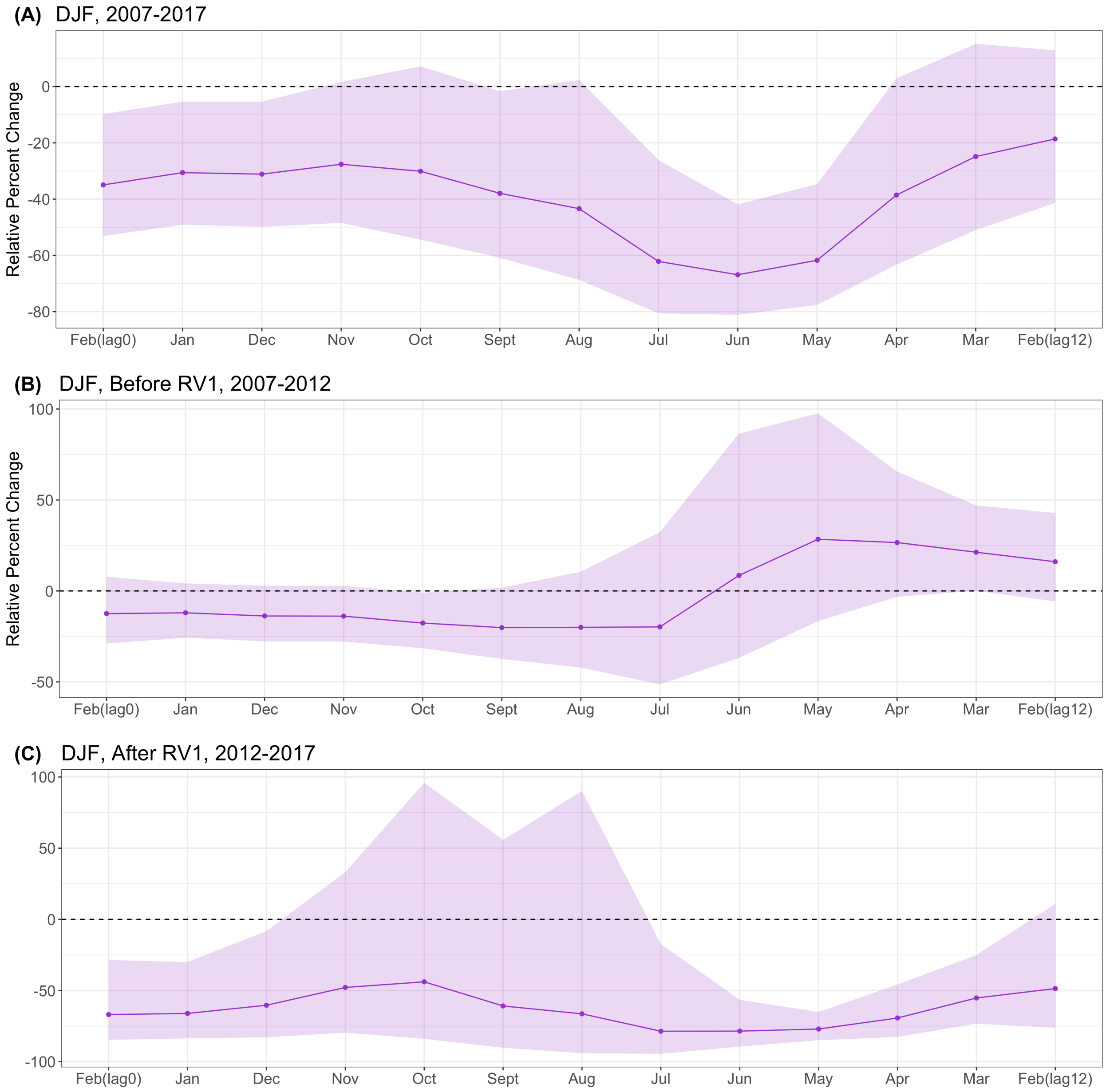

Supplementary Figure 16. December, January, February under-5 diarrhea associated with Niño 4 SST anomalies. The y-axis shows the estimated percent change and 95% confidence intervals in DJF diarrhea incidence associated with 1K increase in Niño 4 SST anomalies. The x-axis represents the month of Niño 4 predictor, which is during or before DJF. Estimates are shown using (A) all of the data (2007-2017), (B) using only data collected before the rotavirus vaccine rollout (Dec. 2007- Feb. 2012), and (C) using only data collected after the rotavirus vaccine rollout (Dec. 2012- Feb. 2017).

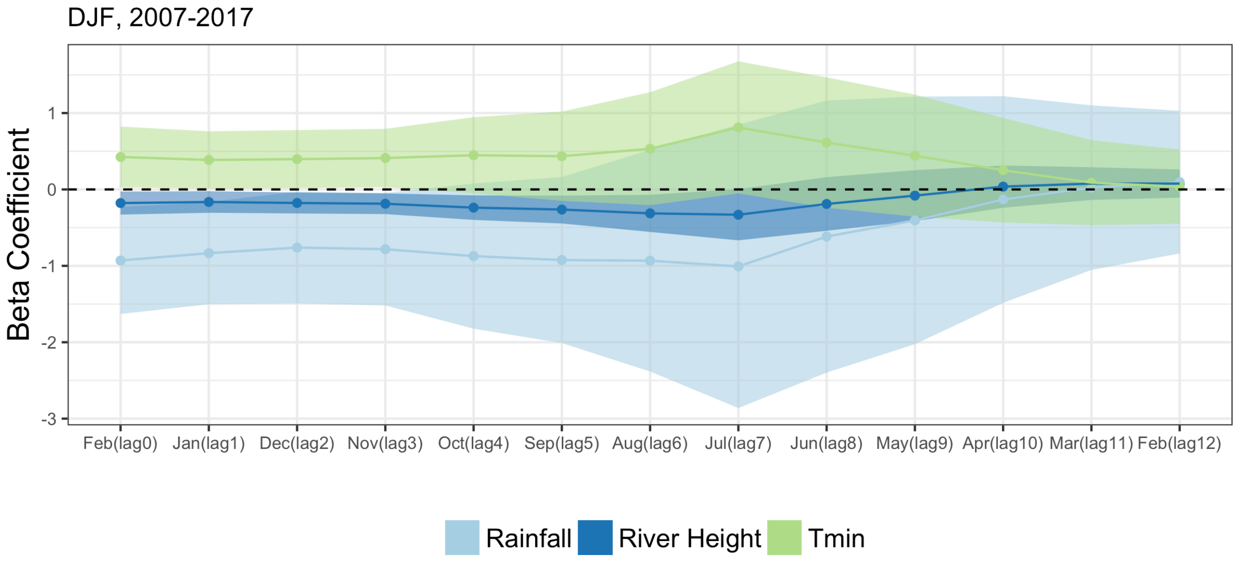

Supplementary Figure 17. December, January, February associations between Niño 4 and environmental variables. Beta coefficients with 95% confidence intervals are shown from regressions predicting total rainfall (light blue, in 100s of millimeters/K), average Chobe River height (dark blue, in meters/K), and average minimum temperature (green, in degrees Celsius/K). Environmental outcomes in DJF were predicted using Niño 4 lagged 0-12 months. The corresponding February from DJF season is lag 0, and the previous February is lag 12. Beta coefficients represent the change in the outcome (in 100s of millimeters for rainfall, meters for river height, and degrees Celsius for temperature) associated with a 1K increase in Niño 4. Regressions were run using data from 2007-2017.

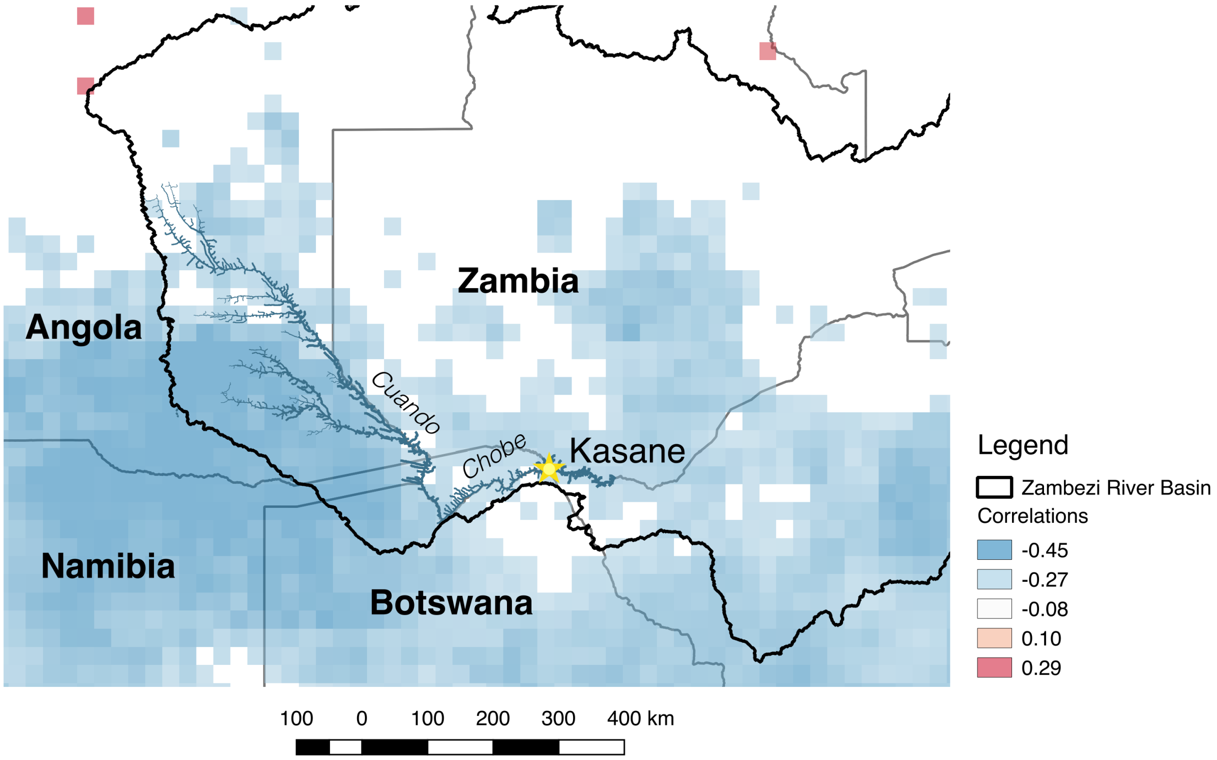

Supplementary Figure 18. Correlations between Niño 4 and TRMM regional rainfall in December, January, February from 1998-2015. Only correlations statistically significant at $p<0.05$ are shown on the map. Blue represents negative correlations and red represents positive correlations. The gold star locates Kasane, which is the largest town in Chobe District, and the black line outlines the Zambezi River Basin.
