# Supplementary material for: El Niño-Southern oscillation and under-5 diarrhea in Botswana
Source: Nat Commun. 2019 Dec 20;10:5798. doi: 10.1038/s41467-019-13584-6 (PMC6925142; doi:10.1038/s41467-019-13584-6)
Supplement: Supplementary file 3 — Reporting Summary [file 41467_2019_13584_MOESM3_ESM.pdf]

nature research

Corresponding author(s): Alexandra K. Heaney

Last updated by author(s): May 28, 2019

Reporting Summary

Nature Research wishes to improve the reproducibility of the work that we publish. This form provides structure for consistency and transparency in reporting. For further information on Nature Research policies, see Authors & Referees and the Editorial Policy Checklist .

Statistics

For all statistical analyses, confirm that the following items are present in the figure legend, table legend, main text, or Methods section.

n/a	Confirmed	
☐	☒	The exact sample size (*n*) for each experimental group/condition, given as a discrete number and unit of measurement
☐	☒	A statement on whether measurements were taken from distinct samples or whether the same sample was measured repeatedly
☐	☒	The statistical test(s) used AND whether they are one- or two-sided<br>*Only common tests should be described solely by name; describe more complex techniques in the Methods section.*
☐	☒	A description of all covariates tested
☐	☒	A description of any assumptions or corrections, such as tests of normality and adjustment for multiple comparisons
☐	☒	A full description of the statistical parameters including central tendency (e.g. means) or other basic estimates (e.g. regression coefficient) AND variation (e.g. standard deviation) or associated estimates of uncertainty (e.g. confidence intervals)
☐	☒	For null hypothesis testing, the test statistic (e.g. *F*, *t*, *r*) with confidence intervals, effect sizes, degrees of freedom and *P* value noted<br>*Give P values as exact values whenever suitable.*
☒	☐	For Bayesian analysis, information on the choice of priors and Markov chain Monte Carlo settings
☒	☐	For hierarchical and complex designs, identification of the appropriate level for tests and full reporting of outcomes
☐	☒	Estimates of effect sizes (e.g. Cohen's *d*, Pearson's *r*), indicating how they were calculated

Our web collection on statistics for biologists contains articles on many of the points above.

Software and code

Policy information about availability of computer code

Data collection	The under-5 diarrhea data and meteorological data were provided by co-author Dr. Kathleen Alexander, who received the data from Botswana Ministry of Health and Department of Meteorological Services. ENSO index data was downloaded from the NOAA website. Hence, no code or specific software was used for data collection.
Data analysis	All data analyses were done using existing packages and code in RStudio (version 1.0.136)

For manuscripts utilizing custom algorithms or software that are central to the research but not yet described in published literature, software must be made available to editors/reviewers. We strongly encourage code deposition in a community repository (e.g. GitHub). See the Nature Research guidelines for submitting code & software for further information.

Data

Policy information about availability of data

All manuscripts must include a data availability statement. This statement should provide the following information, where applicable:

- Accession codes, unique identifiers, or web links for publicly available datasets
- A list of figures that have associated raw data
- A description of any restrictions on data availability

The El Nino-Southern Oscillation datasets analyzed during the current study are available in the NOAA National Climatic Data Center repository, https://www.ncdc.noaa.gov/teleconnections/enso/index.php. TRMM rainfall data analyzed during the current study are available in the NOAA TRMM data repository, https://pmm.nasa.gov/data-access/downloads/trmm. Meteorological data from Chobe District, Botswana are available from the corresponding author by reasonable request. The under-5 diarrhea data that support the findings of this study are available from the Botswana Government Ministry of Health but restrictions apply to the availability of these data, which were used under license for the current study, and so are not publicly available. Researchers may request access to this data directly from the Botswana Government Ministry of Health at http://www.moh.gov.bw/.

Field-specific reporting

Please select the one below that is the best fit for your research. If you are not sure, read the appropriate sections before making your selection.

- [x] Life sciences
- [ ] Behavioural & social sciences
- [ ] Ecological, evolutionary & environmental sciences

For a reference copy of the document with all sections, see nature.com/documents/nr-reporting-summary-flat.pdf

Life sciences study design

All studies must disclose on these points even when the disclosure is negative.

Sample size

In our dataset, each observations represents the number of under-5 diarrhea cases and climate/meteorological conditions in each month across Chobe District. We have monthly observations from January 2007-July 2017, leading to a total of 127 months. For some analyses, we grouped monthly observations into seasons, leading us to have a new sample of 10 years for each seasonal analysis. We obtained as many under-5 diarrhea records as possible from the Botswana Ministry of Health and therefore maximized our sample size. Unfortunately, we can not expand our sample size due to restrictions on availability of health data, but we found statistically significant associations nonetheless. In our manuscript, we report significant associations and suggest that non-significant associations may be due to limited sample size or lack of true association.

Data exclusions

We obtained under-5 diarrhea case counts from 10 health facilities across Chobe District Botswana. We included all facilities in our analyses except for Pandamatenga. We excluded this health facility because residents of Pandamatenga are more reliant on ground water than the residents in the rest of Chobe District (who rely solely on Chobe River surface water). We hypothesized that the relationship between meteorological variability and under-5 diarrhea was linked to changes in Chobe River water quality. Hence, we excluded Pandamatenga because its cases would not be a part of the same environment-disease pathway.

Replication

We did not carry out any experiments, but our results can be replicated using our code.

Randomization

We did not use randomization in our analyses.

Blinding

We did not use blinding in our analyses.

Reporting for specific materials, systems and methods

We require information from authors about some types of materials, experimental systems and methods used in many studies. Here, indicate whether each material, system or method listed is relevant to your study. If you are not sure if a list item applies to your research, read the appropriate section before selecting a response.

Materials & experimental systems

n/a	Involved in the study	
x		Antibodies
x		Eukaryotic cell lines
x		Palaeontology
x		Animals and other organisms
x		Human research participants
x		Clinical data

Methods

n/a	Involved in the study	
x		ChIP-seq
x		Flow cytometry
x		MRI-based neuroimaging
